# An advanced glycation endproduct (AGE)‐rich diet promotes accumulation of AGEs in Achilles tendon

**DOI:** 10.14814/phy2.13215

**Published:** 2017-03-29

**Authors:** Dorthe Skovgaard, Rene B. Svensson, Jean Scheijen, Pernilla Eliasson, Pernille Mogensen, Anne Mette F. Hag, Michael Kjær, Casper G. Schalkwijk, Peter Schjerling, Stig P. Magnusson, Christian Couppé

**Affiliations:** ^1^ Department of Clinical PhysiologyNuclear Medicine & PET and Cluster for Molecular ImagingRigshospitalet and University of CopenhagenCopenhagenDenmark; ^2^ Department of Orthopedic Surgery MInstitute of Sports Medicine and IOC Research Centre CopenhagenBispebjerg Hospital and Center for Healthy AgingFaculty of Health and Medical SciencesUniversity of CopenhagenCopenhagenDenmark; ^3^ Experimental Internal Medicine at the Faculty of HealthMedicine and Life SciencesMaastricht University Medical CenterCopenhagenThe Netherlands; ^4^ Department of Physical TherapyMusculoskeletal Rehabilitation Research UnitBispebjerg HospitalUniversity of CopenhagenCopenhagenDenmark

**Keywords:** Achilles tendon, advanced glycation endproducts, AGE‐rich diet, aging

## Abstract

Advanced Glycation Endproducts (AGEs) accumulate in long‐lived tissue proteins like collagen in bone and tendon causing modification of the biomechanical properties. This has been hypothesized to raise the risk of orthopedic injury such as bone fractures and tendon ruptures. We evaluated the relationship between AGE content in the diet and accumulation of AGEs in weight‐bearing animal Achilles tendon. Two groups of mice (C57BL/6Ntac) were fed with either high‐fat diet low in AGEs high‐fat diet (HFD) (*n* = 14) or normal diet high in AGEs (ND) (*n* = 11). AGE content in ND was six to 50‐fold higher than HFD. The mice were sacrificed at week 40 and Achilles and tail tendons were carefully excised to compare weight and nonweight‐bearing tendons. The amount of the AGEs carboxymethyllysine (CML), methylglyoxal‐derived hydroimidazolone (MG‐H1) and carboxyethyllysine (CEL) in Achilles and tail tendon was measured using ultraperformance liquid chromatography tandem mass spectrometry (UPLC‐MS/MS) and pentosidine with high‐pressure liquid chromatography (HPLC) with fluorescent detection. AGEs in Achilles tendon were higher than in tail tendon for CML (*P* < 0.0001), CEL (*P* < 0.0001), MG‐H1 and pentosidine (for both ND and HFD) (*P* < 0.0001). The AGE‐rich diet (ND) resulted in an increase in CML (*P* < 0.0001), MG‐H1 (*P* < 0.001) and pentosidine (*P* < 0.0001) but not CEL, in Achilles and tail tendon. This is the first study to provide evidence for AGE accumulation in injury‐prone, weight‐bearing Achilles tendon associated with intake of an AGE‐rich diet. This indicates that food‐derived AGEs may alter tendon properties and the development of tendon injuries.

## Introduction

Advanced Glycation Endproducts (AGEs) are chemically modified molecules with pathogenic implications for aging, diabetes and several other chronic diseases (Brownlee 2001; Monnier et al. 2005). AGEs are formed through a nonenzymatic reaction between reducing sugars and free amino groups of proteins, lipids, or nucleic acids. This reaction occurs endogenously in the body, and it is long‐lived proteins in the extracellular matrix (ECM) that are particularly susceptible to AGE modifications with aging (Monnier et al. 2005). In collagen‐rich structures like bone and tendon, AGEs form cross‐links, which morphological modify the collagen network. This alters the biomechanical properties of the tissue with increased stiffness and fragility (Eriksen et al. 1985; Monnier et al. 2005; Zimmermann et al. 2011; Grasa et al. 2013). It has been hypothesized that this accumulation can raise the risk of orthopedic injury such as bone fractures and tendon ruptures (Kjaer 2004; Monnier et al. 2005).

In recent years, much focus has been on the negative effects of dietary intake of AGEs on healthy tissues. Current evidence support that ingestion of AGE‐rich diet increases the AGE content in major arteries, heart, and bone that lead to age‐related changes, and pathology in both animals and humans (Eriksen et al. 1985; Koschinsky et al. 1997; Shapiro et al. 2008; Uribarri et al. 2011).

Previous studies have investigated AGE content and stiffening in nonweight‐bearing rat‐tail tendon (Grasa et al. 2013; Roncero‐Ramos et al. 2014). In line with this, we have recently shown that mice eating chow diet containing markedly higher AGEs N*δ*‐(5‐hydro‐5‐methyl‐4‐imidazolon‐2‐yl)‐ornithine (MG‐H1), N^*ε*^‐(1‐carboxyethyl)lysine (CEL) and N^*ε*^‐(carboxymethyl) lysine (CML) had higher content of AGEs in their nonweight‐bearing tail tendon compared to mice ingesting a fat diet with low amounts of AGEs (Eriksen et al. 1985). The tail tendons of mice with high AGE intake also displayed increased stiffness, which indicates that AGE‐rich diet leads to functionally important accumulation of AGEs in the connective tissue that do not see large loads (Eriksen et al. 1985). However, as tail tendon is only mechanically loaded to a very moderate degree the findings do not reveal whether or not dietary AGE accumulation will be the same in a weight‐bearing and thus more mechanically loaded tendon (Svensson et al. 2016). Recently it was found, that in master athletes the age‐related AGE accumulation in patellar tendon was less pronounced compared to untrained but healthy counterparts (Couppe et al. 2014). Furthermore, mechanical loading during treadmill training in mice increases collagen turnover and reduces AGEs in tibialis anterior tendon (Wood and Brooks 2016). Thus, it is conceivable that weight‐bearing tendons have lower AGE content than less loaded tendons, and we wanted in the present study to investigate whether a AGE‐rich diet promotes AGE accumulation also in tendons that are subjected to high mechanical loading, or whether this mechanical loading rather counteracts the diet induced AGE accumulation.

### Objective/Purpose

The purpose of this study was to investigate if a diet containing high amounts of AGEs (CML, CEL and MG‐H1) results in AGE accumulation in the clinically important Achilles tendon. In addition, we wanted to compare the level of AGEs with data from the previous published study on tail tendon to elucidate possible differences in the quantitative level of AGEs in tendons with different functional importance.

We hypothesized that AGE accumulation would be more pronounced with high dietary AGE intake in Achilles tendon, similarly to that earlier found in the tail tendon. In addition, we hypothesized that the amount of AGEs in Achilles tendon would be lower compared to tail tendon tissue because of a mechanically induced higher collagen turnover in the weight‐bearing Achilles tendon.

## Methods

### Dietary mouse model

This investigation builds on a previously published study, in which atherosclerosis and AGE accumulation in tail tendon tissue were investigated using both homozygous ApoE^−/−^ mice (“atherosclerotic phenotype”) and wild type (WT) mice (C57BL/6Ntac) on different diets (Eriksen et al. 1985). However, in the present substudy we only focused on the impact of the dietary intake of AGEs, and therefore only WT mice were investigated. The mice were obtained at 8 week of age (Taconic Europe, Lille Skensved, Denmark), from which point one‐half of the mice (*n* = 14) were fed ad libitum HFD (protein, 17 cal%; carbohydrate, 43 cal%; fat, 41 cal%; Western Diet, #D12079B, Research Diets, New Brunswick, NJ) to induce obesity, and the other half (*n* = 11) were fed ad libitum normal diet (ND) (protein, 27 cal%; carbohydrate, 60 cal%; fat, 13 cal%; #1310, Altromin Spezialfutter & KG, Germany). The difference of the group size is due to loss of tissue samples. As previously published, the amount of AGEs were 6‐50 times higher in ND compared to HFD (ND vs. HFD: CML: 206.1 vs. 4.7 nmol/g; CEL: 86.5 vs. 3.0 nmol/g; MG‐H1: 554.0 vs. 34.5 nmol/g;) (Eriksen et al. 1985).

Mice were sacrificed at 40 weeks of age by decapitation after having fasted overnight and being anesthetized for a period of 4 h, during which time the mice were ^18^F‐Fluoro‐Deoxy‐Glucose Positron Emission Tomography (PET) scanned as part of the main study looking at the progression of atherosclerosis (Hag et al. 2012). The hind legs of each mouse were removed and stored at −20°C until the experimental day. One experienced researcher (PE) carefully excised the Achilles tendon *in toto*, being conscientious not to include muscle fibers in the sample using a technique that was performed in previous studies (Eliasson et al. 2007, 2009).

Animal care and experimental procedures were performed under the approval of the Animal Experiments Inspectorate in Denmark (permit number 2011/561‐14).

### AGE analysis

Each Achilles tendon sample was incubated in 1 mL of 1 mol/L NaCl at 4°C for 24 h with at least one fluid exchange. After removal of NaCl, the tendon samples were incubated in 1 mL of 0.5 mol/L acetic acid at 4°C for 24 h with one fluid exchange. The acetic acid was removed and the tendon samples were incubated with 1 mL of chloroform‐methanol (2:1 vol/vol) at 4°C for 24 h with one fluid exchange (Eriksen et al. 1985; Dunn et al. 1991). After removal of the organic solvent, the tendon samples were dried under a gentle stream of nitrogen at room temperature. To avoid AGE formation during hydrolysis, samples were reduced with 0.2 mL of 0.1 mol/L NaBH_4_ in 0.2 mol/L borate buffer (pH 9.2) for 2 h at room temperature. The reduced samples were deproteinized in 1 mL of cold (4°C) 20% (vol/vol) trifluoracetic acid and centrifuged at 4300*g*, 4°C for 20 min, and the supernatant was discarded. The remaining pellets were hydrolyzed in 0.5 mL of 6 mol/L HCl. From each hydrolysate, 40 *μ*L were taken and mixed with 20 *μ*L of internal standard before analysis. CML, CEL, and MG‐H1 were analyzed by ultraperformance liquid chromatography tandem mass spectrometry (UPLC‐MS/MS) while pentosidine was analyzed using high pressure liquid chromatography (HPLC) fluorescence as described earlier(Scheijen et al. 2009; Hanssen et al. 2013). Hydroxyproline (hyp) was analyzed by UPLC‐MS/MS. In short, a dilution of the hydrolysate was mixed with internal standard trans‐4‐hydroxy‐l‐proline‐2, 5,5‐d_3_ (D3‐hyp) and dried under a gentle stream of nitrogen at 70°C. The residue was dissolved in 1 mL of water‐acetonitrile (1:9, vol/vol) and injected onto a hydrophilic interaction chromatography (HILIC) UPLC column (Acquity UPLC BEH HILIC, 1.7 *μ*m, 2.1 × 50 mm). Solvent A was 10 mmol/L ammonium formate‐acetonitrile (1:9, vol/vol) and Solvent B was 10 mmol/L ammonium formate‐acetonitrile (5:5, vol/vol). A linear gradient was started at 95% Solvent A, which was changed to 60% Solvent A within 2 min. After the column with 100% Solvent B was cleaned for 1.5 min, the column was equilibrated for 4 min to the initial conditions. Injection volume was 0.2 *μ*L (partial loop injection) at a column temperature of 45°C. Hyp and D3‐hyp were detected in multiple‐reaction monitoring electrospray positive mode (MRM‐ESI) at a capillary voltage of 0.25 kV, a cone voltage of 25 V, and a desolvation temperature of 600°C (Acquity UPLC, Xevo TQ MS; Waters, Milford, MA). Quantification of hyp was performed by calculating the peak area ratio of hyp (MRM, 132.0 > 86.0) to the internal standard D3‐hyp (MRM, 135.0 > 89.0). The levels of AGEs were expressed as nanomoles of AGE per millimole hyp.

AGEs in Achilles tendon were assessed at a later date than the tail tendon. To rule out the possibility that the observed difference could be due to time difference in assessment, small amounts of spare tail tendon and Achilles tendon tissue were dissolved in papain and assessed for AGE‐specific fluorescence. Here, the Achilles tendon samples also showed significantly higher fluorescence than the tail tendons, when assessed at the same time (data not shown).

### Data reduction and statistics

Results were analyzed using a two‐way mixed model ANOVA (tissue type, diet) with repeated measures for the tissue type since Achilles and tail tendons were from the same animal. Initially the analysis included interactions, if the interaction was not significant, data were finally analyzed using only main effects. Tukey method for multiple comparisons was used in post hoc tests.

## Results

### Baseline characteristics

As previously reported (Eriksen et al. 1985), mice fed with HFD had higher body weight compared to mice consuming ND (HFD: 47.4 ± 3.6 vs. ND: 33.9 ± 3.4 gram, *P* < 0.0001), but there were no significant differences in plasma glucose (HFD: 15.8 ± 5.5 vs. ND: 13.3 ± 4.8 mmol/L, *P* = 0.35) or cholesterol level (HFD: 15.9 ± 5.5 vs. ND: 12.0 ± 4.2 mmol/L, *P* = 0.104).

### Advanced glycation end products

**Figure 1:**
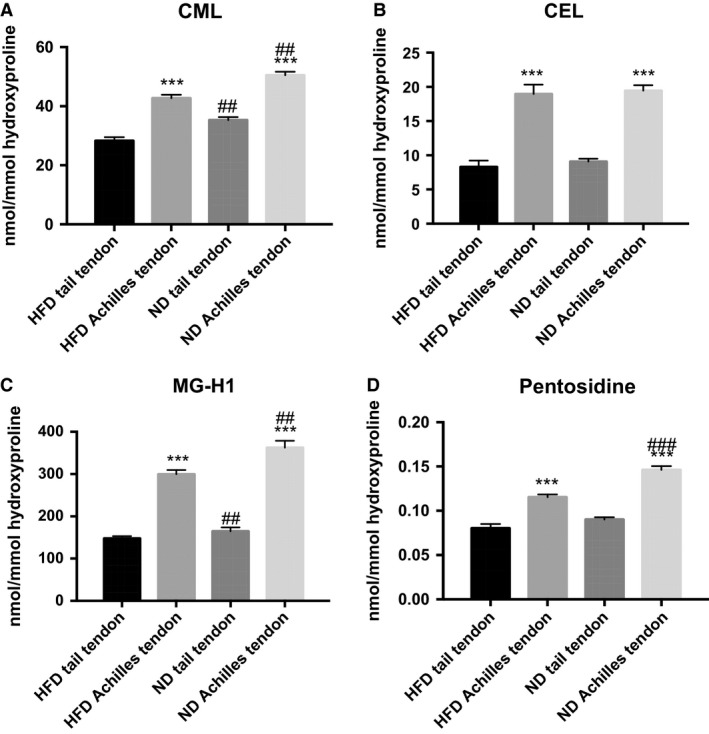
Advanced glycation endproduct content in tail tendon and Achilles tendon. Data given as Mean ± SEM, Fat: *n* = 11, Chow: *n* = 14, (A): CML: ***diff from tail *P* < 0.0001 (main effect), ##diff from HFD *P* < 0.001 (main effect). (B): CEL: ***diff from tail *P* < 0.0001 (main effect) (C): MG‐H1: ***diff from tail *P* < 0.0001 (main effect), ## diff from HFD *P* < 0.001 (main effect). (D) Pentosidine: There was an interaction (*P* < 0.01), which showed an increase in pentosidine for the ND group in the Achilles (### diff from HFD *P* < 0.0001), but not in the tail tendon (*P* = 0.27). For both diets there was an effect of tissue (***diff from tail *P* < 0.0001). HFD, high‐fat diet.

Data and effect sizes are shown in Figure 1 and Table 1, respectively (see below). There were more AGEs in the Achilles tendon than the tail tendon for CML (*P* < 0.0001) (Fig. 1A), CEL (*P* < 0.0001) (Fig. 1B) and MG‐H1 (*P* < 0.0001) (Fig. 1C) for both ND and HFD. This was also the case for pentosidine in both the ND (*P* < 0.0001) and HFD (*P* < 0.0001) group (Fig. 1D).

**Table 1:** Biochemical parameters

	Effect of Achilles tendon	Effect of ND
CML (nmol/mmol hydroxyproline)	14.9 ± 0.7***	7.4 ± 1.4**
CEL (nmol/mmol hydroxyproline)	10.5 ± 0.9***	0.6 ± 0.9
MG‐H1 (nmol/mmol hydroxyproline)	177.4 ± 13.6***	39.6 ± 10.1**
Pentosidine (within Tail) (nmol/mmol hydroxyproline)		0.010 ± 0.005
Pentosidine (within Achilles) (nmol/mmol hydroxyproline)		0.031 ± 0.005***
Pentosidine (within ND) (nmol/mmol hydroxyproline)	0.056 ± 0.005***	
Pentosidine (within HFD) (nmol/mmol hydroxyproline)	0.035 ± 0.005***	

Mean ± SE for each group. Mean difference for effects of Achilles tendon and ND. Significant difference (**P < 0.001, ***P < 0.0001).

For pentosidine there was an interaction (*P* < 0.01), which showed a significant increase in pentosidine for the ND group in the Achilles (*P* < 0.0001), but not in the tail tendon (*P* = 0.27). For both diets there was an effect of tissue (*P* < 0.0001).

HFD, High‐Fat Diet; ND, Normal Diet.

The ND led to increased CML (*P* < 0.0001) (Fig. 1A) and MG‐H1 (*P* < 0.001) (Fig. 1C) in both the Achilles and tail tendons. CEL in the tendons was not significantly affected by ND (Fig. 1B). Except for pentosidine, none of the measured AGEs displayed any significant interaction term in the ANOVA, indicating that the effect of diet was identical for the Achilles and tail tendons. For pentosidine there was an interaction (*P* < 0.01) (Fig. 1D), which showed an increase in pentosidine for the ND in the Achilles (*P* < 0.0001) but not in the tail tendon (*P* = 0.27).

## Discussion

To the best of our knowledge, this is the first study to provide evidence of AGE accumulation in Achilles tendons only as a function of intake of an AGE‐rich diet compared to high‐fat (but low AGE) diet, thereby extending our previous findings with dietary AGEs as an important source of AGE accumulation in tendon tissue. Furthermore, contrary to our hypothesis, Achilles tendon demonstrated greater AGE content (CML, MG CEL and pentosidine) than tail tendon. This is somewhat surprising, as mechanical loading earlier has been shown to reduce accumulation of AGE in the weight‐bearing patellar tendon (Kongsgaard et al. 2009; Couppe et al. 2014).

Tendon mechanical properties are determined by the underlying composition and structure, although details still remains to be described. Type I collagen is the primary components of tendon ECM, constituting up to 85% of the tissue dry mass (Kjaer 2004; Svensson et al. 2016). Covalent, intermolecular cross‐links associated with the assembly of the collagen fibrils are believed to be a major source of mechanical strength, and improved mechanical properties during development are likely related to maturation of these enzymatic cross‐links (Eyre 1987; Bailey et al. 1998). However, the slow turnover of collagen allows for the accumulation of AGEs with aging that reduce collagen fibril sliding leading to impaired tendon mechanics (Verzijl et al. 2000; Li et al. 2013; Fessel et al. 2014). Although the biomechanical properties were not investigated in the present study, others and we have previously reported, that AGE‐rich diet leads to AGE accumulation and increased mechanical properties (mainly in the region of plastic deformation, where AGEs might play a role) in non weight‐bearing tail tendons (Eriksen et al. 1985; Roncero‐Ramos et al. 2014). In line with the study hypothesis, the present data provides evidence of an AGE‐rich diet leading to AGE accumulation in Achilles tendon and thereby has an aging effect on tissue as previously reported (Eriksen et al. 1985). Consequently, AGE deposition in tendons might contribute to the higher incidence of tendinopathy and tendon rupture, as was already suggested in patients with diabetes and with aging (Kjaer 2004; de Jonge et al. 2011, 2015; Ranger et al. 2016; Svensson et al. 2016). However, future studies will have to address this issue also in normal individuals of different ages without diabetes.

Our second hypothesis was that AGE‐content would be lower in Achilles tendon compared to tendon tail, irrespective of the diet, due to a presumed load‐induced higher collagen turnover (Wood and Brooks 2016). Interestingly, the present data does not support this hypothesis.

An obvious explanation is that AGE cross‐linking is the result of a balance between exposure and rates of collagen synthesis/turnover, with the half‐life of collagen being one important determinant (Baynes 2000). Thorpe et al. found a clear difference in AGE content in two weight‐bearing equine tendons with aging (Thorpe et al. 2010). The high strain and injury‐prone superficial digital flexor tendon (comparable to the human Achilles tendon) displayed a higher AGE content and much lower collagen turnover (estimated half‐life of collagen 197 years) compared to the low strain and rarely injured common digital extensor tendon, which had an estimated collagen half‐life of 34 years (Thorpe et al. 2010). Our data therefore indicates that Achilles tendon, despite being subjected to greater loading, has a lower collagen turnover than tail tendon allowing larger amounts of AGEs to accumulate. The Achilles tendon is the largest tendon in the body and is very important during locomotion. It is possible that a low collagen turnover is necessary for optimal integrity and function of this important tendon. In fact, human Achilles tendon collagen fibrils (core part of tendon) have been found to be practically inert throughout adult life (Heinemeier et al. 2013).

An important aspect is to what extent AGE accumulation can be counteracted with regular exercise/loading of the tissue. We recently demonstrated that individuals with type 2 diabetes have similar AGE content (pentosidine) as healthy controls in their Achilles tendons although they have a markedly higher AGE content in the skin (Couppe et al. 2016). These data suggest that weight‐bearing connective tissue may respond differently to glycemic loading than nonweight‐bearing connective tissue. It has been suggested that mechanical loading can increase collagen turnover and thereby prevent AGE accumulation (Avery and Bailey 2005; Kongsgaard et al. 2009; Couppe et al. 2014). In support of this, life‐long running and resistance training have shown to reduce the age‐associated accumulation of the AGE level in the human patellar tendon (Kongsgaard et al. 2009; Couppe et al. 2014). It is therefore possible that mechanical strain of the tendon and/or just a general systemic effect (better glycemic control) associated physical activity could help offset age‐induced accumulation of AGE in tendons (Odetti et al. 1996; Boor et al. 2009). It is also possible that AGE‐related pathogenic processes and the aging effect on tissue including those of tendons may be influenced by dietary AGE restriction. Several recent studies have implicated that low AGE‐diet can reduce AGEs (pentosidine) in rodent tail tendon tissue and on whole‐body level lower the markers of oxidative stress, inflammation and insulin resistance (Sell and Monnier 1997; Lingelbach et al. 2000; Vlassara et al. 2016). In the present study, the difference in AGE content is likely related to lower carbohydrate (sugar) content in the HFD and the processing of the two types of diet, where ND was manufactured with heating, which is well known to induce AGEs (Maillard reaction) (Maillard 1912; Uribarri et al. 2010).

We cannot exclude the possibility that other differences between the diets (ND vs. HFD), for example, carbohydrate concentration, macronutrients and total energy intake, also contributed to the AGE accumulation in the tendons of the present study. However, others have demonstrated that with AGE‐rich but isocaloric diet intake, pathologic changes in serum and tissue are still induced (Cai et al. 2007). Of note, dicarbonyl stress caused by the most reactive AGE precursor methylglyoxal (MGO) measured in this study can without sugar such as glucose or without going through the classical Maillard reaction pathways induce AGE formation (Rabbani and Thornalley 2012). Inflammation per se may also form AGEs that again induce inflammation creating a vicious cycle (Hanssen et al. 2014). The study was limited by a small sample size that possibly led to failure to demonstrate a significant difference in pentosidine for the tail tendon, which was actually demonstrated in our previous report (Eriksen et al. 1985).

In conclusion, we have found evidence for an accumulation of AGEs in the weight‐bearing Achilles tendon due to intake of AGE‐rich diet. This suggests that food‐related intake of AGEs could potentially alter tendon properties and thereby represent a risk factor in the development of tendon injuries.

## Conflict of Interest

None declared.
